# Ion‐Exchange‐Induced Phase Transition Enables an Intrinsically Air Stable Hydrogarnet Electrolyte for Solid‐State Lithium Batteries

**DOI:** 10.1002/advs.202310005

**Published:** 2024-04-04

**Authors:** Chenghao Cui, Fan Bai, Yanan Yang, Zhiqian Hou, Zhuang Sun, Tao Zhang

**Affiliations:** ^1^ State Key Lab of High‐Performance Ceramics and Superfine Microstructure Shanghai Institute of Ceramics Chinese Academy of Sciences 1295 Dingxi Road Shanghai 200050 P. R. China; ^2^ Center of Materials Science and Optoelectronics Engineering University of Chinese Academy of Sciences Beijing 100049 P. R. China; ^3^ CAS Key Laboratory of Materials for Energy Conversion Shanghai Institute of Ceramics Chinese Academy of Sciences 1295 Dingxi Road Shanghai 200050 P. R. China

**Keywords:** air stability, composite electrolyte, garnet electrolytes, Li‐H exchange reaction, solid‐state batteries

## Abstract

Inferior air stability is a primary barrier for large‐scale applications of garnet electrolytes in energy storage systems. Herein, a deeply hydrated hydrogarnet electrolyte generated by a simple ion‐exchange‐induced phase transition from conventional garnet, realizing a record‐long air stability of more than two years when exposed to ambient air is proposed. Benefited from the elimination of air‐sensitive lithium ions at 96 h/48e sites and unobstructed lithium conduction path along tetragonal sites (12a) and vacancies (12b), the hydrogarnet electrolyte exhibits intrinsic air stability and comparable ion conductivity to that of traditional garnet. Moreover, the unique properties of hydrogarnet pave the way for a brand‐new aqueous route to prepare lithium metal stable composite electrolyte on a large‐scale, with high ionic conductivity (8.04 × 10^−4^ S cm^−1^), wide electrochemical windows (4.95 V), and a high lithium transference number (0.43). When applied in solid‐state lithium batteries (SSLBs), the batteries present impressive capacity and cycle life (164 mAh g^−1^ with capacity retention of 89.6% after 180 cycles at 1.0C under 50 °C). This work not only designs a new sort of hydrogarnet electrolyte, which is stable to both air and lithium metal but also provides an eco‐friendly and large‐scale fabrication route for SSLBs.

## Graphical abstract

In this work, an intrinsically air‐stable hydrogarnet electrolyte is developed by the elimination of the air‐sensitive lithium‐ions at octahedral sites (96h/48e) without significantly sacrificing the ionic conductivity. Additionally, hydrogarnet‐reinforced polyethylene oxide composite electrolytes (CPEs) are also provided based on a water‐based route, avoiding the stringent drying environment required by traditional garnet‐based CPEs.

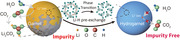

## 1 Introduction

Solid‐state lithium batteries (SSLBs) are believed to outperform the current mainstream commercial lithium‐ion batteries due to the utilization of solid‐state electrolytes (SSEs) with intrinsic non‐flammability, good compatibility with lithium metal, and outstanding electrochemical stability.^[^ ^1^ ^]^ Consequently, the development of SSEs has attracted significant attention from both industry and academia, with new advances focusing on ionic conductivity improvement,^[^ ^2^ ^]^ lithium dendrite suppression,^[^ ^3^ ^]^ and interfacial compatibility^[^ ^4^ ^]^ emerging continually in recent years. However, the unsolved intrinsic chemical instability of almost all SSEs developed toward either lithium or air has greatly inhibited material production and consequently the development of SSLBs.^[^ ^5^ ^]^ In the group of oxide electrolytes, garnet‐type Li_6.5_La_3_Zr_1.5_Ta_0.5_O_12_ (LLZTO) electrolyte possesses relatively high ionic conductivity (>1 mS cm^−1^) and excellent stability toward lithium metal and a wide electrochemical window (>5 V, experimental measured).^[^ ^6^ ^]^ Despite these advantages, the practical application of garnet electrolyte has been hindered by its relatively insufficient stability under ambient air. Under the guideline of conductivity improvement, Li‐stuffed garnet structures (Li7) with abundant lithium ions at 96h(48e) sites have been developed based on the original Li3 and Li5 garnet.^[^ ^7^ ^]^ However, when in contact with H_2_O and CO_2_, those highly reactive 96h(48e) lithium ions spontaneously reacted to the generation of lithium conduction insulation byproducts such as LiOH, Li_2_CO_3_, and La_2_Zr_2_O_7_ and finally to the structural crack by internal stress.^[^ ^8^ ^]^

In consideration of improving the air stability of LLZTO, multiple strategies such as doping (Ga, Ca co‐doping et. al) and coating (LiCoO_2_ by lithium donor reaction et al.) have been developed.^[^ ^9^ ^]^ By tuning the occupancy of lithium ions at octahedral sites (96 h or 48e) or directly constructing stable shells on LLZTO particles, the air stability of LLZTO could gradually increase to several days. Nevertheless, the kinetically favored Li–H exchange reaction could not totally be avoided as long as a large number of lithium ions still occupy octahedral sites. Instructively, some research focusing on the Li–H exchange found that lithium ions at 96 h sites can be easily substituted by protons without structure collapse and pointed out that the protonate process seems to have a thermodynamic limit.^[^ ^10^ ^]^ These results inspired us that by rational designing Li–H exchange reaction, deep protonated garnet electrolytes with excellent air stability can be acquired. In this work, via a pre‐ion‐exchange reaction, we fully pined highly reactive 96h(48e) sites with thermodynamic stable protons and obtained a deep pronated hydrogarnet electrolyte with a space group (SG) transition from I a $\bar{3}$ d to I $\bar{4}$ 3d. The hydrogarnet electrolyte not only realized the intrinsic air stability of garnet‐type LLZTO but also maintained the lithium‐ion migration paths based on air‐stable lithium ions at tetragonal sites.

To experimentally validate the efficacy of this strategy, long‐time stability tests were conducted, revealing that the hydrogarnet remained stable even after prolonged exposure to ambient air for two years. Impurities such as Li_2_CO_3_ could hardly be detected in hydrogarnet powder. This super‐stability stems from the absence of air‐sensitive lithium ions, rendering the hydrogarnet electrolyte intrinsically resistant to both water and carbon dioxide in ambient air, which was further verified by first‐principal calculations. Moreover, acceptable ionic conductivity is retained in the hydrogarnet structure with a lithium‐ion conduction path incorporating 12a sites and vacancies. The 3D interconnected lithium migration network is visualized by band‐valence simulation, and a relatively low lithium‐ion migration activation energy (0.43 eV) is substantiated through both theoretical and experimental analysis. More compellingly, a polyethylene oxide (PEO) based composite electrolyte incorporating hydrogarnet was fabricated via aqueous solution in ambient air. This environment‐friendly composite electrolyte boasts an ionic conductivity of 8.04 × 10^−4^ S cm^−1^ (50 °C), a lithium‐ion transference number of 0.43, and a wide electrochemical window of 4.95 V. Leveraging this composite electrolyte, the constructed SSLBs also demonstrated low polarization, an extended cycle life, and superior rate performance.

## 2 Results and Discussions

### 2.1 Compositional and Structural Alteration of Hydrogarnet

**Figure 1:**
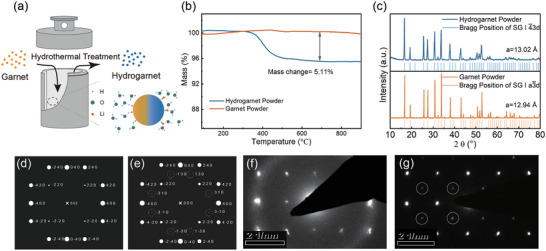
a), the scheme illustrating the process of converting garnet to hydrogarnet via the Li–H reaction under hydrothermal conditions; b), TG curve of both garnet and hydrogarnet electrolyte powder in the range of 100–900 °C; c), XRD patterns of both garnet and hydrogarnet, and the vertical bars representing the allowed Bragg positions of both SG I $\bar{4}3$ d and SG I a $\bar{3}$ d; the simulated SEAD patterns of d) garnet structure and e) hydrogarnet structure alone the (001) axis; the corresponding experimentally obtained SEAD patterns of f) garnet electrolyte powder and h) electrolyte powder alone the (001) axis.

First of all, the straightforward approach for transforming garnet into air‐stable hydrogarnet was presented. This transformation was based on a typical Ta‐doped garnet electrolyte LLZTO of SG I a $\bar{3}$ d, which was prepared using a solid‐state reaction route. To substitute the highly reactive lithium ions at 96h(48e) sites with immobilized H^+^, as illustrated by **Figure** **1**a, the pristine garnet powder was subjected to a hydrothermal treatment to kinetically enable the exchange at both the 24d and 96h(48e) sites. The morphological change after hydrothermal treatment was initially explored. From the scanning electron microscope (SEM) and energy dispersion energy (EDS) images (Figures S1 and S2, Supporting Information), no obvious morphological change and compositional segregation is observed under this elaborately designed reaction condition (impact of reaction conditions is illustrated in Figures S3 and S4, Supporting Information). To meticulously track the transformation from garnet powder to hydrogarnet, analysis including inductively coupled plasma optical emission spectrometer (ICP‐OES) tests and thermogravimetry analysis (TG) was conducted. The ICP‐OES results reveal that after hydrothermal treatment, the lithium content of the resultant powder is reduced by 77%, while the concentrations of heavy cations in the garnet structure remain virtually unchanged (Table S1, Supporting Information). This implies that H^+^ likely enter the garnet lattice to maintain electric neutrality. In the TG curve (Figure 1b), the hydrated garnet electrolyte exhibits a 5.11% mass loss at temperatures exceeding 500 °C. Meanwhile, only La_2_Zr_2_O_7_ and the garnet phase are detected in the X‐ray diffraction (XRD) result of the residues of calcination (Figure S5, Supporting Information), attributing the weight loss primarily to the release of H^+^ in the form of H_2_O. To demonstrate the structural evolution from garnet to hydrogarnet, XRD tests were carried out. As depicted in Figure 1c, the diffraction peaks in the XRD pattern of pristine garnet electrolyte aligns well with the Bragg positions of SG I a $\bar{3}$ d. In the pattern of the hydrogarnet, almost all peaks of garnet are retained but the emergence of a tiny new peak at 21.4°, indicates a structural divergence. This peak cannot be simply explained by the SG I a $\bar{3}$ d or secondary phases but fits well with the Bragg position of the plane (310) in SG I $\bar{4}$ 3d, suggesting that a “hydrogarnet” phase of SG I $\bar{4}$ 3d generated due to the intense Li‐H exchange.^[^ ^11^ ^]^ Meanwhile, as shown in Figure 1c, the lattice parameter of hydrogarnet is 13.02 Å, which is also larger than that of pristine garnet (12.94 Å) and relates to proton substitution.^[^ ^12^ ^]^ This result is also supported by the measured d_002_ of hydrogarnet in the high‐resolution transmission electron microscope images (Figure S6, Supporting Information). To further confirm the structural evolution of the hydrogarnet electrolyte, selected area electron diffraction (SAED) analysis was employed, leveraging the larger scattering factor (Table S2, Supporting Information) of the electron beam compared to X‐ray. Since no reference SAED pattern for hydrogarnet is available, simulated SAED patterns of both the pristine garnet and the hydrogarnet are presented in Figure 1d,e. From the simulated patterns along the [100] zone axis, obvious distinctions can be observed between these two structures. In the original garnet structure of SG I a $\bar{3}$ d, diffraction spots of (310) and (510) lattice planes are absent. Conversely, in the hydrogarnet structure of SG I $\bar{4}$ 3d, the diffraction spots of lattice planes (310) and (510), although very weak (labeled by dashed cycles), can be distinguished. This distinction arises from the structural extinction rules: in SG I a $\bar{3}$ d structure, (hk0) planes must

satisfy the condition h, *k* = 2n to avoid structural extinction, whereas in the non‐centrosymmetric SG I $\bar{4}$ 3d structure, the reflection condition of (hk0) alters to h + k = 2n. This theoretical result gives support to the experimentally acquired SAED patterns shown in Figure 1e,f. Despite the presence of secondary diffraction interference (Figure S7, Supporting Information),^[^ ^13^ ^]^ the anticipated diffraction spots of (310) and (510) are observed in the experimental SAED pattern of I $\bar{4}$ 3d (labeled by solid cycles) while are absent in that of I a $\bar{3}$ d, conclusively validating the formation of the hydrogarnet phase.

### 2.2 Superior Air Stability of the Hydrogarnet

**Figure 2:**
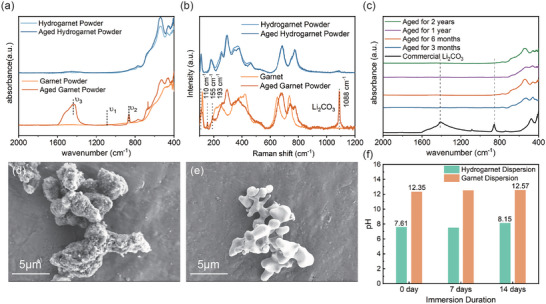
a) FT‐IR spectra and b) Raman spectra of garnet and hydrogarnet powder before and after aged in the air for one month; c) FT‐IR spectra of the garnet electrolyte after exposure to air for different durations; d) SEM images of garnet electrolyte particles and e) hydrogarnet electrolyte particles aged for two years; f) The pH values of the suspension of garnet and hydrogarnet and its variation over time.

Subsequently, the superior stability of the hydrogarnet electrolyte was experimentally verified for a period of more than two years. To demonstrate the superior air stability of hydrogarnet, Fourier transform infrared spectroscopy (FT‐IR) and Raman spectroscopy analyses were conducted on both pristine garnet and hydrogarnet powders after a one‐month exposure to air. The FT‐IR spectrum of the

one‐month‐aged garnet electrolyte (**Figure** **2**a) reveals a distinctive dual absorption band near 1500 cm^−1^, indicative of the asymmetric tensile vibration of ─C═O (υ3). This observation, coupled with the weak band at 1100 cm^−1^ and a narrow band at 860 cm^−1^, supports the formation of Li_2_CO_3_.^[^ ^14^ ^]^ In stark contrast, the FT‐IR spectrum of the aged hydrogarnet remains almost unchanged, suggesting the outstanding stability of hydrogarnet. This finding is further corroborated by the Raman spectra (Figure 2b), which display a strong band at 1100 cm^−1^ and weak bands at 193, 155^,^ and 110 cm^−1^, aligning well with commercial Li_2_CO_3_ but only discernible in garnet.^[^ ^15^ ^]^ To further prove the exceptional air stability of the hydrogarnet, FT‐IR analyses (Figure 2c) of samples exposed to ambient air for extended periods (6 months, 1 year, and 2 years) were consecutively performed. Notably, even after two years of exposure to ambient air, the hydrogarnet maintains its crystal structure unchanged, and the presence of Li_2_CO_3_ in the hydrogarnet is negligible, making this as the best air stability result to our knowledge. (Figure S8, Supporting Information). To more vividly verify this deduction, SEM images were obtained and presented in Figure 2d,e. The pristine garnet is fully covered with fluffy impurities primarily composed of lithium carbonate, while the hydrogarnet powder maintains its smooth surface and shape after being exposed to the air for two years. Moreover, an extreme test involving the dispersion of both pristine garnet and hydrogarnet in water was conducted to assess their stability under rigorous conditions, as garnet's severe Li–H exchange is known to cause a rapid pH increase. Dispersions of both hydrogarnet and garnet powders in deionized water with a solid content of 5 wt.% were prepared and the pH values were continuously monitored. As demonstrated in Figure 2f, the pH of the freshly prepared garnet powder dispersion surges to 12.3, indicating strong basicity. In contrast, the pH of the as‐prepared hydrogarnet electrolyte dispersion remains neutral at 7.6 throughout the entire observation period of over two weeks, further attesting to the outstanding stability of hydrogarnet toward water. Additionally, the hydrogarnet electrolyte exhibits remarkable stability against common organic solvents, as evidenced in Figures S9 and S10 (Supporting Information).^[^ ^16^ ^]^

### 2.3 Mechanism of Superior Air Stability of the Hydrogarnet

**Figure 3:**
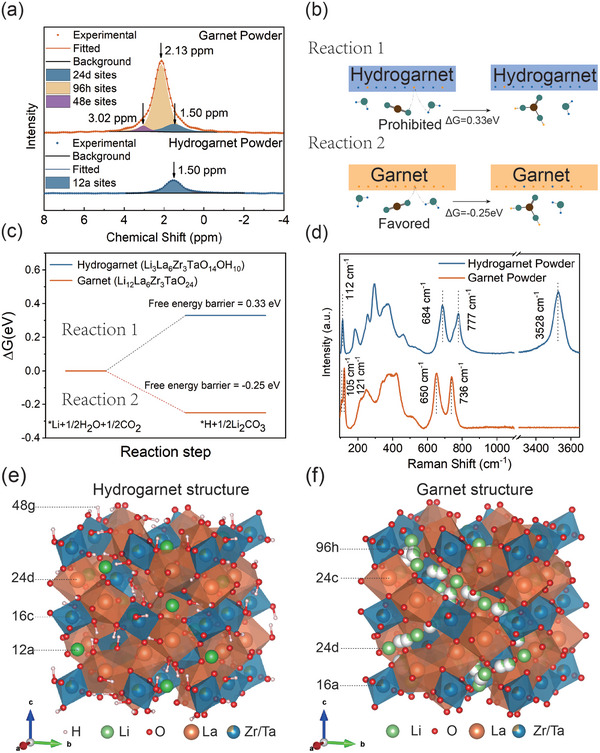
a) the ^6^Li MAS NMR spectra of both garnet and hydrogarnet electrolytes; b) the schemes of our proposed degradation process of both garnet and hydrogarnet electrolytes and c) the corresponding free Gibbs energy changes; d) Raman spectra of both garnet and hydrogarnet electrolyte; e) the schemes of crystal structures of hydrogarnet and garnet f) structure. The schemes were drawn with the VESTA software.^[^ ^20^ ^]^

The stability of hydrogarnet to ambient air and even water is highly related to its unique structure. Given that both oxygen and heavy metal ions inherit the same position in the original garnet structure, the key to the excellent air stability of hydrogarnet lies in the re‐distribution of lithium ions. In this part, we provide detailed chemical environment information on Li^+^ and H^+^ to further understand their distribution.^[^ ^17^ ^]^ Through the magic angle spinning nuclear magnetic resonance (MAS NMR) test, the chemical environment of lithium ions is demonstrated. As shown in **Figure** **3**a, The ^6^Li MAS NMR signal of pristine garnet powder can be decoupled by 3 Gaussian‐Lorentzian mixed bands^[^ ^18^ ^]^ centered at +1.50 ppm, +2.13 ppm, and +3.02 ppm. These bands correspond to the 24d, 96 h, and 48e sites of the Ta‐doped garnet electrolyte respectively, which is in accordance with previous reports.^[^ ^19^ ^]^ Conversely, in the hydrogarnet's ^6^Li MAS NMR spectrum, only a single band at +1.50 ppm is observed, correlating to the 24d sites in the original garnet. This suggests a complete extraction of lithium ions from the 96 h and 48e sites in the hydrogarnet electrolyte. Notably, considering that 77% of lithium ions are extracted, merely 12 out of the original 56 lithium ions remain within the hydrogarnet unit cell, occupying half of the original 24d sites. Besides, owing to the loss of centrosymmetry, the 24d sites in the garnet structure would transform into 12a and 12b sites in the hydrogarnet structure, and the chemical environment is distinct between these two sites because of the interruption of protons (described subsequently). It can be seen that around the 12b site, there are four protons surrounding it while the protons are absent around the 12a site (Figure S11, Supporting Information). Considering the electrostatic repulsion of the protons around the 12b site, Li‐ions in hydrogarnet mainly occupy the 12a site and possess the same chemical coordination environment as the original 24d site in garnet, fitting well with ^6^Li MAS NMR results.^[^ ^11^ ^]^ Lithium ions in hydrogarnet situated at the 12a sites are tightly enclosed at the center of the tetrahedron, contributing to their exceptional chemical stability. It is only in this fully protonated hydrogarnet that superior air stability is realized. For comparison, a partially protonated garnet electrolyte was synthesized via conventional acid treatment, and its stability was also assessed (Figure S12, Supporting Information). This partially protonated garnet failed to retain its stability in the air even after a mere two‐week duration. This instability was also manifested in the powder that underwent hydrothermal treatment at low temperatures, as evidenced by the obtained FT‐IR spectra (Figure S13, Supporting Information).

Based on the structure resolved above, density functional theory (DFT) calculations were employed to deepen the understanding of the air stability of the hydrogarnet electrolyte. These calculations were tailored to examine the reaction kinetics of lithium ions positioned at various sites within the garnet and hydrogarnet structures, assuming the typical lithium‐ion extracted from a whole garnet or hydrogarnet unit cell to react with H_2_O and CO_2_. As illustrated in Figure 3b,c, when lithium ions at the 12a sites in hydrogarnet structure participate in the formation of lithium carbonate (Equation 1), the Gibbs free energy change of this system is +0.33 eV, proving the degradation of hydrogarnet is thermodynamically prohibited, indicating the intrinsic air stability of hydrogarnet. For lithium ions at 48e sites in garnet, this reaction (Equation 2) lead to a Gibbs free energy change of −0.25 eV, suggesting a thermodynamically spontaneous degradation process.

(1) $$\begin{array}{ccc} & & Li_{12}La_{24}Zr_{12}Ta_{4}O_{56}OH_{40}+\frac{1}{2}H_{2}O+\frac{1}{2}CO_{2}\\ & & \to Li_{11}La_{24}Zr_{12}Ta_{4}O_{55}OH_{41}+\frac{1}{2}Li_{2}CO_{3},\;\Delta G\;=\;+0.33\;\mathrm{eV} \end{array}$$

(2) $$\begin{array}{ccc} & & Li_{52}La_{24}Zr_{12}Ta_{4}O_{96}+\frac{1}{2}H_{2}O+\frac{1}{2}CO_{2}\to Li_{51}H_{1}La_{24}Zr_{12}Ta_{4}O_{96}\\ & & +\;\frac{1}{2}Li_{2}CO_{3},\;\Delta G\;=\;-0.25\;\mathrm{eV} \end{array}$$

The distribution of protons is then discussed here to provide a further understanding of the hydrogarnet structure and the unique distribution of Li‐ions. Most conspicuously, the Li–H exchange process introduces significant changes observable in the Raman spectra (Figure 3d). Specifically, a broad band ≈3400 cm^−1^ associated with hydroxyl emerges in the hydrogarnet structure, suggesting that the H^+^ ions in the structure are anchored by oxygen to form hydroxyls. This is also supported by the broad peak at +4 ppm demonstrated in the ^1^H projection of the ^1^H ^7^Li HETCOR MAS NMR spectrum of the hydrogarnet (Figure S14, Supporting Information), which is consistent with the MAS NMR signals of hydroxyl in similar compounds.^[^ ^21^ ^]^ In the ^1^H‐^7^Li HETCOR MAS NMR result, a clear signal correlating the single ^7^Li band with the ^1^H band is observed. This strong ^1^H‐^7^Li dipolar coupling indicates that the H^+^ ions enter the hydrogarnet structure and are situated in relatively close spatial positions to the Li^+^ ions distributed alone the 12a‐12b‐12a migration pathes.^[^ ^22^ ^]^ To rational demonstrate the positioning of H^+^ in hydrogarnet, DFT‐based structural relaxation was implemented, by initially placing H^+^ at the 48e sites (Figure S15, Supporting Information).^[^ ^23^ ^]^ The optimized proton positions align closely with the above conclusions where Li^+^ occupying the 12a sites, Zr^4+^ (Ta^5+^) occupying the 16c sites, La^3+^ occupying the 24d sites, and proton occupying the 48 g sites in Figure 3e. This arrangement presents notable differences compared to the original structure of the garnet electrolyte depicted in Figure 3f.

### 2.4 Ionic Conductivity Performance of the Hydrogarnet

**Figure 4:**
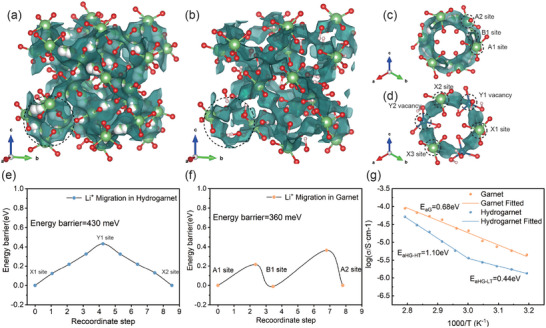
a) BVEL maps of garnet and b) hydrogarnet structure in general; c) BVEL maps of the lithium migration paths parallel to the (111) axis of both garnet and d) hydrogarnet (enlargement of the section encircled by the dashed line in (a) and (b)); e) calculated energy profiles for Li^+^ migration alone the designed route in hydrogarnet structure and f) garnet structure using CENEB method via DFT calculations; g) the experimental acquired ionic conductivity of garnet and hydrogarnet electrolyte powder under different temperature via a cold‐pressed method.

The ion conduction of the hydrogarnet was performed considering both structural and compositional transformations from garnet to hydrogarnet. Initially, to understand how lithium conduction in hydrogarnet compares to that in garnet, a band‐valance energy landscape (BVEL) calculation was conducted.^[^ ^24^ ^]^ The BVEL analysis provides a visual representation of the lithium migration paths, allowing for direct observation of the effects of proton substitution and structure alteration. In the original garnet of I a $\bar{3}$ d, a well‐interconnected lithium migration network alone 24d‐48e(96 h)−24d paths is distinctly demonstrated in **Figure** **4**a,c. Upon transitioning to the hydrogarnet of SG I $\bar{4}$ 3d, as illustrated in Figure 4b,d, the migration paths between 12a sites gradually shrink but remain unobstructed, owning to the occupation of protons. To provide a theoretical quantification of the energy barrier of lithium diffusion of hydrogarnet, the climbing image nudged elastic band (CINEB) method based on DFT calculations was utilized.^[^ ^25^ ^]^ In the garnet electrolyte, the calculated profiles (Figure 4e) indicate that following the trajectories of lithium ions outlined by the BVEL map, the activation energy barrier (*E_a_*) for lithium‐ion hopping from A1 (24d site) ‐to B1(48e site)‐ to A2 (24d site) is 360 meV (the higher barrier at B1‐A2 route results from the occupancy of Li‐ion at 96 h site partially blocking the path), which is in accordance with previous reports.^[^ ^26^ ^]^ Considering the partial occupation of protons in the 48e sites of the hydrogarnet structure (40 protons occupying 83.3% of the 48e sites), two lithium‐ion migration trajectories with different proton occupations are selected and illustrated in Figure 4d. For the hydrogarnet structure, the *E_a_* of Li^+^ hopping alone X1(12a site)‐Y1(12b site)‐X2(12a site) route is 430 meV (shown in Figure 4f). Along this route, the lithium is hypothesized to traverse a 48 g vacancy with three proton occupations. When Li^+^ migrates along the X2(12a site)‐Y2(12b site)‐X3(12a site) route passing through a 48 g vacancy with four proton occupations, the *E_a_* increased to 550 meV (shown in Figure S16, Supporting Information). Compared to the garnet electrolyte, the increased *E_a_* is only 70 (or 190 meV), indicating that the lithium migration in hydrogarnet is not drastically impeded.

In addition to the theoretical insights from BVEL and DFT calculations, the lithium conduction properties were also investigated experimentally. Considering the restraint of its insufficient thermal stability, hydrogarnet cannot be sintered into bulk pellets. Therefore, the ionic conductivities of hydrogarnet and garnet were measured in powder form using a cold‐pressed cell configuration (illustrated in Figure S17, Supporting Information). It is worth noting that ionic conductivity based on the cold‐pressed method may be lower than the ideal values by one to two orders of magnitude because of the large interparticle resistance. The method is still insightful and crucial for providing a qualitative comparison.^[^ ^27^ ^]^ Figure 4g shows the Arrhenius plots of the hydrogarnet and garnet electrolytes acquired by the electrochemical impedance spectroscopy (EIS) technique. As expected, accompanied by the loss of lithium and additional occupation of protons, lithium conduction of the hydrogarnet electrolyte decreases inevitably. Specifically, at 30 °C, ion conduction of the hydrogarnet electrolyte (1.34 × 10^−6^ S cm^−1^) is reduced to 30% of the garnet electrolyte (4.47 × 10^−6^ S cm^−1^). The experimentally measured *E_a_* of hydrogarnet electrolyte is 0.44 eV near ambient temperature, which is quite close to the theoretical obtained value (0.43 eV). However, it should be noted that the high ionic conductivity of garnet electrolyte results from its ultra‐high lithium concentration (39.88 mol L^−1^), while for hydrogarnet, the lithium concentration decreases to 9.2 mol L^−1^. After normalization to lithium concentration, per mole of lithium ions in the hydrogarnet electrolyte even exhibit higher specific ionic conductivity (1.12 × 10^−7^ S cm^2^ mol^−1^ vs 1.45 × 10^−7 ^S cm^2^ mol^−1^) than the garnet electrolyte. Moreover, an unusual transition of activation energy is observed above 60 °C, which might be associated with the activation of proton migration at a higher temperature, as indicated by a quasi‐elastic neutron scattering test done by X.M. Liu et. al.^[^ ^28^ ^]^ Overall, both experimental and theoretical findings confirm that acceptable lithium conduction is preserved in hydrogarnet electrolytes, highlighting their potential for application in electrochemical energy storage devices.

### 2.5 Water‐Based Fabrication of Hydrogarnet Composite Electrolyte

**Figure 5:**
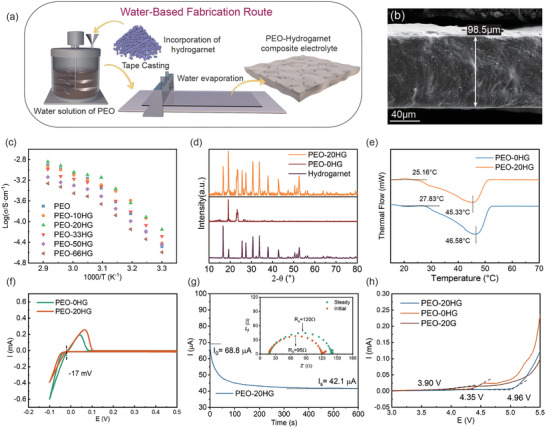
a) A scheme of the water‐based fabrication process of the PEO‐xHG composite electrolyte; b) cross‐sectional SEM images of PEO‐20HG electrolyte; c) XRD patterns of PEO‐20HG, PEO‐0HG and hydrogarnet powder; d) ionic conductivities of PEO‐xHG (x = 10, 20, 33, 50, 66 wt.%) under different temperature; e) DSC curves of both PEO‐0HG and PEO‐20HG electrolyte; f) CV curves of the Li‐Li symmetry cells assembled with PEO‐0HG and PEO‐20HG electrolyte in the range of −0.1–0.5 V; g) *I–t* curves of the Li–Li symmetry cell assembled with PEO‐20HG electrolyte before and after polarization, and the inset showed the corresponding Nyquist plots; h) LSV plots of PEO‐0HG, PEO‐20G and PEO‐20HG electrolyte.

The excellent air stability could functionalize hydrogarnet to both novel and large‐scale applications in solid‐state batteries. As illustrated in **Figures** **5**a and S18 (Supporting Information), based on the superior stability of hydrogarnet toward H_2_O and CO_2_, a PEO‐based composite electrolyte (CPE) reinforced by hydrogarnet electrolyte powders was designed and produced via an original aqueous route under ambient air. From the SEM images (Figure 5b; Figure S18, Supporting Information), it can be seen that the uniformly dispersed hydrogarnet is successfully integrated into the PEO matrix, forming a PEO‐hydrogarnet composite electrolyte with a thickness ≈100 µm (98.5 µm). To ascertain the enhancement of hydrogarnet powder on electrolyte performance, ionic conductivities of the composite electrolyte containing different amounts of hydrogarnet (labeled as PEO‐xHG, x = 10, 20, 33, 50, 66 wt.%) at different temperatures were measured. From Arrhenius plots (Figure 5c; Figure S19, Supporting Information), the PEO‐20HG CPE exhibits the highest ionic conductivity (7.02 × 10^−5^ S cm^−1^ at 30 °C and 8.04 × 10^−4^ S cm^−1^ at 50 °C) and lower migration activation energies compared to PEO‐0HG at both 50 °C (0.16 eV compared to 0.19 eV) and 30 °C (0.44 eV compared to 0.47 eV). Although the incorporation of a proper amount of hydrogarnet can reduce the crystallinity of PEO and create interparticle Li‐ion pathways to improve ionic conductivities, the compatibility of hydrogarnet electrolyte filler with the water‐based CPE preparation process is the prerequisite for these improvements. As shown in XRD patterns (Figure 5d), only peaks of hydrogarnet and PEO‐LiTFSI matrix are detected in the composite electrolyte, and impurities such as Li_2_CO_3_ are absent, proving the stability of hydrogarnet during the water‐based preparation process. This stability is further verified by the FT‐IR results. For comparison, FT‐IR tests on CPEs containing 20 wt.% pristine garnet (PEO‐20G) prepared via the same process was conducted. The presence of characteristic bands of Li_2_CO_3_ and the significantly degraded ionic conductivities (Figures S20 and S21, Supporting Information) indicate the detrimental impact of impurities on the garnet surface or dissolved in CPEs. In contrast, hydrogarnet remains stably dispersed in deionized water for over 24 h, thanks to the interaction between the abundant hydroxyl exposed on the particle surface and water molecules(Figure S22, Supporting Information).^[^ ^29^ ^]^ Benefiting from both excellent stability and dispersion, hydrogarnet leads to a notable enhancement in the ionic conductivity of CPEs. In addition, the exposed hydroxyls may coordinate with the oxygen atom in the PEO chain segment, reducing the crystallinity of PEO and weakening the strength of the Li^+^‐O coordination in PEO.^[^ ^30^ ^]^ As illustrated in differential scanning calorimetry (DSC) curves (Figure 5e), the onset temperature and the peak temperature of PEO‐20HG (25.16 and 45.33 °C) are lower than those (27.83 and 46.58 °C) of PEO‐0HG. Combining a larger full width at half maxima of PEO‐20HG peaks shown in XRD results (Figure S23, Supporting Information), a weaker crystallinity of PEO is confirmed. And as an active filler, hydrogarnet powders create interparticle Li^+^ transport pathways in the CPE, thereby contributing to ionic conductivity.^[^ ^31^ ^]^ Despite the ionic conductivity of PEO‐xHG decreases with higher hydrogarnet content due to the intense scattering of Li‐ion transportation by large amounts of hydrogarnet‐PEO interfaces, at a relatively high hydrogarnet content of 66 wt.%, no drastic reduction (from 9.07 × 10^−4^ S cm^−1^ to 3.5 × 10^−4^ S cm^−1^ at 60 °C) in total conduction of CPE is observed (Figure 5c). However, replacing the 66 wt.% hydrogarnet with inert zirconia oxide filler results in a significant decrease in total ion conduction (2.4 × 10^−5^ S cm^−1^ at 50 °C, Figure S24, Supporting Information). This high ionic conductivity of PEO‐66HG can be attributed to the establishment of interparticle Li‐ion transport pathways between hydrogarnet fillers even with a high solid content, further indicating the intrinsic ionic conductivity of hydrogarnet electrolyte.

**Figure 6:**
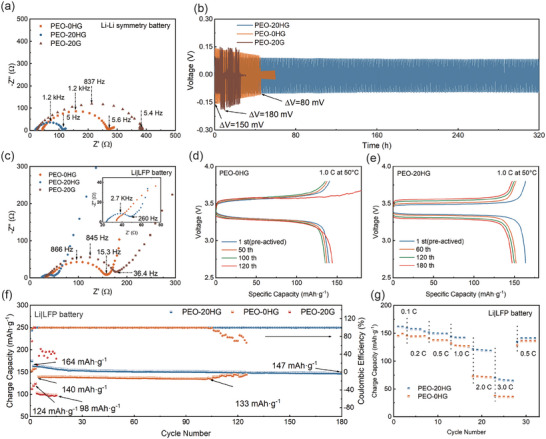
a) the Nyquist plots and b) galvanostatic cycling performance of the Li‐symmetry batteries assembled with PEO‐0HG, PEO‐20HG and PEO‐20G electrolytes; c) the Nyquist plots of the Li|LFP batteries assembled with PEO‐0HG, PEO‐20HG and PEO‐20G electrolytes, and the inset is an enlargement of the Nyquist plots of the Li|LFP battery with PEO‐20HG; d) the charge and discharge curves of Li|LFP batteries assembled with PEO‐0HG and e) PEO‐20HG at different cycles; f) the cycling performance of the Li‐LFP batteries assembled with PEO‐0HG, PEO‐20HG and PEO‐20G electrolytes at the rate of 1.0C; g) the rate performance for Li|LFP batteries assembled with PEO‐0HG and PEO‐20HG electrolytes. All electrochemical tests were carried out under 50 °C in an air‐blowing thermostatic oven.

For a more comprehensive assessment of the electrochemical performance of hydrogarnet CPEs, detailed electrochemical tests are presented below. Cyclic voltammetry (CV) between −0.1–0.5 V in Figure 5f reveals only one reduction peak corresponding to lithium deposition at −0.017 V (v.s. Li^+^/Li), indicative of the electrochemical stability of PEO‐20HG to lithium metal anode. This compatibility with lithium metal anode makes hydrogarnet electrolyte outperform other water‐stable Li‐ion conductor fillers (e.g., LATP and LLTO). Further, the linear sweep voltammetry (LSV) plot demonstrates that PEO‐20HG does not exhibit any oxidation current until 4.95 V (Figure 5h), whereas the PEO‐0HG electrolyte shows a clear oxidation current peak at 4.34 V. This improved electrochemical stability of PEO‐20HG not only suggests that hydrogarnet reinforces the stability of PEO, but also indicates that hydrogarnet itself remains stable against electrochemical oxidation up to 4.95 V. In sharp contrast, a significant oxidation current is observed in PEO‐20G at 3.90 V, which can be attributed to the pristine garnet surface impurities in the air. Additionally, the lithium‐ion transference numbers for both PEO‐0HG and PEO‐20HG electrolytes are investigated using potentiostatic polarization and the *I–t* curves (Figure 6g; Figure S25, Supporting Information). Calculated by the formula shown in the method section, the lithium‐ion transference number of PEO‐20HG is 0.43, which is obviously higher than that of the PEO‐0HG electrolyte (0.18).

### 2.6 Electrochemical Performance of SSLBs with PEO‐HG Composite Electrolyte

Encouraged by the promising characterizations of hydrogarnet, the performance of SSLBs with hydrogarnet‐based CPEs was further evaluated. Li–Li symmetry batteries were individually assembled with PEO‐20HG, PEO‐20G, and PEO‐0HG and subjected to cycling tests at 50 °C with a current density of 0.2 mA cm^−2^. Profiting from the reduction in crystallinity resulting from the compatibility of hydrogarnet in CPEs mentioned above, the cell impedance for batteries with PEO‐20HG is greatly minimized (100 Ω cm^2^ compared to 250 Ω cm^2^ of PEO‐0HG and 380 Ω cm^2^ of PEO‐ 20G, shown in **Figure** **6**a), indicating an improved interfacial contact. Besides, charge‐discharge overpotential is also reduced (90 mV) compared to that using filler‐free PEO‐0HG electrolyte (140 mV) or pristine garnet PEO‐20G electrolyte (180 mV). Meanwhile, the cycle life also confirms the effectiveness of the corporation of hydrogarnet. Lithium symmetry battery with PEO‐20HG operates steadily for more than 300 h at a current density of 0.2 mA cm^−2^ (Figure 6b) and for 500 h at a current density of 0.1 mA cm^−2^ (Seen from Figure S26, Supporting Information). Conversely, batteries based on the PEO‐0HG electrolyte and PEO‐20G electrolyte confront short circuits after operating 100 and 28 h at a current density of 0.2 mA cm^−2^, respectively. Additionally, the overpotential in batteries with PEO‐20HG electrolyte remains relatively stable throughout the cycling process, whereas in batteries with PEO‐0HG electrolyte, the overpotential consistently decreases. This observation implies the emergence and development of potential micro‐shorts caused by lithium dendrite may occur in batteries with PEO‐0HG electrolyte but is insignificant to that of PEO‐20HG during the cycling process, suggesting an improved lithium dendrite suppression of PEO‐20HG electrolyte.

To investigate the electrochemical performance of PEO‐20HG, PEO‐20G, and PEO‐0HG electrolytes in SSLBs, coin cells with a lithium metal anode and a LiFePO_4_ (LFP) cathode were assembled and tested. First, the EIS tests were conducted at 50 °C to acquire the overall impedance of SSLBs. As shown in Figure 6c, The Li|LFP battery using PEO‐20HG electrolyte shows the lowest total resistance of 50 Ω cm^2^ prominently lower than those using PEO‐0HG electrolyte (157 Ω cm^2^) and PEO‐20G electrolyte (177 Ω cm^2^). As a consequence, the Li||LFP battery with PEO‐20HG electrolyte has a small overpotential (148 mV) and a higher specific capacity (164 mAh g^−1^) at 1.0C (Figure 6d). Impressively, even after 180 cycles, the overpotential remains below 275 mV. In contrast, the Li|LFP battery with PEO electrolyte has a significantly higher overpotential (300 mV) and a lower specific capacity (140 mAh g^−1^) (seen in Figure 6d,e). After 120 cycles under this significant polarization, the PEO‐0HG begins to decompose, resulting in erratic capacities and diminished coulombic efficiency, highlighting PEO's instability under high polarization.^[^ ^32^ ^]^ Owning to the poor electrochemical stability, PEO‐20G CPEs in Li|LFP batteries confront severe decomposition at a rate of 1.0C (Figure S27, Supporting Information). The Li|LFP battery using PEO‐20HG electrolyte can operate stably for 200 cycles at 1.0C with a capacity retention rate of 90.2% (Figure 6f). However, the Li|LFP battery using PEO‐0HG electrolyte only operates stably for 120 cycles at 1.0C, and the coulombic efficiency shows a drastic drop after cycling for 120 times, corresponding to the instability shown in Figure 6d. Disappointingly, the Li|LFP battery with PEO‐20G electrolyte could not sustain stable operation even in the first cycle at a rate of 1.0C, exhibiting extremely low coulombic efficiency. Figure 6g illustrates that, owing to a slower interfacial resistance and a higher lithium transference number, the rate performance of the Li|LFP battery with PEO‐20HG electrolyte (160 mAh g^−1^ at 0.1C, 141 mAh g^−1^ at 1.0C and 65 mAh g^−1^ at 3.0C) surpasses that of the PEO electrolyte (161 mA h g^−1^ at 0.1C, 126 mAh g^−1^ at 1.0C and 35 mAh g^−1^ at 3.0C). Additionally, lower overpotentials are also observed in the Li|LFP battery with PEO‐20HG electrolyte (Figure S28, Supporting Information). While batteries based on PEO‐20G cannot even withstand a rate of 1.0C, rendering reliable rate performance unattainable. These above results underscore the promising prospect of the utilization of PEO‐HG CPEs as well as hydrogarnet electrolytes in the realm of SSLBs.

## 3 Conclusion

A novel hydrogarnet electrolyte was designed and obtained by subjecting the garnet electrolyte to an intense ion exchange (Li–H exchange) reaction under hydrothermal treatment. This meticulously crafted hydrogarnet electrolyte showcases marvelous stability for two years in ambient air and maintains its stability even when immersed in water. The underlying mechanism is attributed to the alteration in the distribution of lithium ions in the hydrogarnet structure. Unlike the garnet structure where numerous lithium‐ions occupy the air‐sensitive 96 h/48e site, hydrogarnet positions lithium‐ions at the 12a site and is firmly enclosed in the center of the tetrahedron, bestowing itself with intrinsic air stability. Despite a much lower lithium concentration, hydrogarnet only exhibits a marginal decrease in ionic conductivity (with 30% retained compared to the garnet) and a minor increase in lithium‐ion migration activation energy (from 0.39 to 0.43 eV). When normalized to lithium concentration, each mole of lithium ions in the hydrogarnet electrolyte contributes to a higher ionic conductivity. Profiting from the outstanding stability of hydrogarnet toward water, a water‐based fabrication of composite electrolyte (paired with water‐soluble PEO) was developed. The resulting PEO‐20HG CPE demonstrates an improved ionic conductivity (8.04 × 10^−4^ S cm^−1^ vs 6.87 × 10^−4^ S cm^−1^ at 50 °C), an enlarged electrochemical window (4.95 V vs 4.38 V), and a higher lithium transference number (0.43 vs 0.18) compared to the PEO‐0HG electrolyte. The SSLBs with PEO‐20HG CPE exhibit better interfacial contact and enhanced lithium dendrite suppression, thus possessing higher specific capacity (157 mAh g^−1^), lower overpotential (148 mV), extended cycle life (180 cycles at 1.0C), and better rate performance. This work introduces an innovative approach to enhancing the intrinsic chemical stability of garnet‐like electrolytes and heralds a new era for the advancement of eco‐friendly oxide‐based SSLBs.

## 4 Experimental Section

See the Supporting Information for details of the materials and methods.

## Conflict of Interest

The authors declare no conflict of interest.

## Supporting information

Supporting Information

## Data Availability

The data that support the findings of this study are available from the corresponding author upon reasonable request.
