# Supplementary material for: Ion‐Exchange‐Induced Phase Transition Enables an Intrinsically Air Stable Hydrogarnet Electrolyte for Solid‐State Lithium Batteries
Source: Adv Sci (Weinh). 2024 Apr 4;11(22):2310005. doi: 10.1002/advs.202310005 (PMC11165529; doi:10.1002/advs.202310005)
Supplement: Supplementary file 1 — Supporting Information [file ADVS-11-2310005-s001.pdf]

ADVANCED SCIENCE Open Access

Supporting Information

for *Adv. Sci.*, DOI 10.1002/advs.202310005

Ion-Exchange-Induced Phase Transition Enables an Intrinsically Air Stable Hydrogarnet Electrolyte for Solid-State Lithium Batteries

Chenghao Cui, Fan Bai, Yanan Yang, Zhiqian Hou, Zhuang Sun and Tao Zhang**

Supporting Information

Ion-Exchange-Induced Phase Transition Enables an Intrinsically Air-stable Hydrogarnet Electrolyte for Solid-State Lithium Batteries

Chenghao Cui[a][b], *Fan Bai*[a]*, *Yanan Yang*[a], *Zhiqian Hou*[a], *Zhuang Sun*[a], *Tao Zhang*[a] [b] [c]*

[a]. Dr. C. Cui, Dr. F. Bai, Dr. Y. Yang, Dr. Z. Hou, Dr. Z. Sun, Prof. Tao Zhang
State Key Lab of High-Performance Ceramics and Superfine Microstructure, Shanghai Institute of Ceramics, Chinese Academy of Sciences, 1295 Dingxi Road, Shanghai, 200050, P. R. China
E-mail: taozhang@mail.sic.ac.cn, baifan@mail.sic.ac.cn
[b]. Dr. C. Cui, Prof. Tao Zhang
Center of Materials Science and Optoelectronics Engineering, University of Chinese Academy of Sciences, Beijing 100049, P. R. China
[c]. Prof. Tao Zhang
CAS Key Laboratory of Materials for Energy Conversion Shanghai Institute of Ceramics, Chinese Academy of Sciences, 1295 Dingxi Road, Shanghai, 200050, P. R. China.

Methods

Synthesis of garnet and hydrogarnet electrolyte

The garnet electrolyte powder $Li_{6.5}La_3Zr_{1.5}Ta_{0.5}O_{12}$ was synthesized via a solid-state route reported by previous works[1]. To get the deep hydrated hydrogarnet electrolyte powder, hydrothermal treatment was utilized. Firstly, garnet electrolyte powder was ultrasonically dispersed in deionized (DI) water with a content of 10 wt.%. Then, the garnet electrolyte dispersion was transferred to stainless steel autoclaves with polytetrafluoroethylene (PTFE) lining, and the sealed autoclave was placed in an oven and heated to the target temperature for 6 hours. It is worth noting that the applied hydrothermal treatment temperature should not exceed 373K to avoid the generation of impurities such as $La(OH)_3$. After cooling down naturally, the hydrogarnet electrolyte powder was finally collected by centrifugation. After washing with deionized (DI) water until the obtained filtrate was neutral, hydrogarnet electrolyte powder was obtained and dried at 80°C in an oven for 12 h.

Structural and compositional characterization methods

To detect the phase composition, XRD (X-ray diffraction) tests were conducted with a Bruke D8 Advanced X-ray diffractometer. The morphology and the EDS (energy dispersive spectrometer) mapping of garnet powder, hydrogarnet powder, and PEO-xHG CPEs were conducted by a Zeiss Sigma 300 field emission scanning electron microscopy (SEM). The high-resolution TEM images and SEAD patterns of garnet and hydrogarnet electrolytes were obtained by FEI Tecnai G2 F20 TEM. Raman spectroscopy tests of garnet and hydrogarnet were conducted by a Renishaw inVia Raman spectrometer. Fourier-transform infrared (FT-IR) test results were obtained by Thermal Fisher Nicolet iS5 FT-IR spectrometer. The pH values of both garnet dispersion and hydrogarnet dispersion were obtained by a Horiba LAQUA F71G pH meter. The ^6Li magic angle spinning nuclear magnetic resonance (MAS NMR) tests were carried out with a Bruker Advanced NEO NMR spectrometer.

The ^1H-^7Li heteronuclear correlation (HETCOR) MAS NMR experiment was carried out on a Bruker Avance III HD 500 M spectrometer (11.7 T). The resonance frequencies of ^1H and ^7Li are 500.1 MHz and 194.4 MHz respectively. Spectra were recorded at room temperature using a 4-mm Bruker magic angle spinning (MAS) probe at a spinning speed of 12 kHz. The TPPM15 pulse sequence used during the sampling period corresponds to a 160° pulse length of 5.3 us and a relaxation time of 1 s. The nutation frequency corresponding to the ^1H cross-polarization ramp energy is reduced from 36.2 kHz to 18.1 kHz. The optimized nutation frequency of ^7Li is 53.2 kHz, and the cross-polarization contact time is 1.25 ms. The chemical shifts of ^1H and ^7Li were referenced to H_2O (= 4.7 ppm) and 1 M LiCl solution (= 0 ppm).

The aging process was conducted under full open ambient air for up to 2 years. The electrolyte powder was placed in Petri dishes and the Petri dishes were positioned inside a rack with only two sides and a top, to prevent dust from falling into the powder. After aging for 6 months, 1 year, and 2 years, part of the aged electrolyte powder was used for compositional and structural tests.

Theoretical calculation methods

All first-principles calculations are performed in the VASP software[2] using projection augment-wave (PAW)[3] Ab initio with periodic boundary conditions in the framework of density functional theory (DFT). The exchange-correlation energy is calculated using the generalized gradient approximation (GGA) of Perdew-Burke-Ernzerh of (PBE)[2] . These systems are assumed to be paramagnetic, and standard spin-polarized DFT calculations were performed throughout the study. Monkhorst-Pack grid is performed using 3×3×1 for structural optimization, and 9×9×1 is used for electronic calculations. The convergence criteria for the total energy and atomic forces are 10^{-5} eV and 10^{-4} eV·Å, respectively, within the above parameters. The minimum energy paths and the energy barriers for K-ion dissociation were obtained by the climbing image nudged elastic band (CINEB) method. [4]

The free energy (ΔG) of each reduction step was obtained at zero bias potential using,[5]

$$\Delta G = \Delta E + \Delta E_{ZPE} - T\Delta S$$

where ΔE was the reaction energy, ΔE_{ZPE} was the difference in zero-point energies, T was the temperature (298.15K) and ΔS is the reaction entropy.

The simulated SEAD was obtained by the ReciPro software package based on the CIF (crystallographic information file) optimized by DFT calculations. An electron beam with an accelerated voltage of 200 keV was used in the simulation. Band-valence energy landscape calculations were performed based on Bond_Str software using the same CIF files as SEAD simulations.[6] The mesh density was 80×80×80, and the d-max was selected to 5.0 angstrom. The activation energy of the iso-surface shown in BVEL maps is 1.5 eV.

Fabrication of PEO-hydrogarnet composite electrolyte

Firstly, 660 mg Polyethylene oxide (PEO, Aladdin, Mw=600 000) and 286 mg Bis(trifluoromethane)sulfonimide lithium salt (LiTFSI, Aladdin, 99.99%) were dissolved in 5.65 mL deionized water to get a homogeneous PEO-LiTFSI solution. Then different amounts of nano-sized hydrogarnet powder (0, 25 mg, 50 mg, 100 mg, 200 mg, or 400 mg) were added to 2 mL PEO-LiTFSI solution, and the mixture was magnetically stirred for 12 hours to form a uniform slurry. After being cast into PTFE molds. the slurry was dried at

50°C under ambient air for 6 hours. Finally, the composite electrolyte was obtained after vacuum drying at 60°C for 12 hours.

Cell assembly and electrochemical measurements

In order to facilitate ion transportation inside the cathode, a composite $LiFePO_4$ (LFP) cathode containing PEO and LiTFSI was utilized. Firstly, 65 mg PEO and 35 mg LiTFSI were dissolved in 3 mL DI water. Then, 800 mg LFP powder (carbon coated, Shenzhen Dynanonic Co., Ltd.) and 100 mg Super P-Li (TIMCAL Graphite & Carbon Co., Ltd.) were added into the solution to form the cathode slurry. After magnetically stirring for 12 h, the slurry was tape-casted to alumina foil with a 150 μm blade, and the Al foil was heated under 60°C for 2 hours to get rid of water solvent. Finally, LFP composite cathodes were obtained after vacuum drying for 12 hours and the mass loading was 2-3 mg·cm^{-2}

The Li|LFP batteries and lithium symmetry batteries were assembled within CR2032-type coin cells. The Li-symmetry batteries were fabricated by attaching two lithium sheets (Φ12mm) to both sides of the CPEs. The Li|LFP batteries were fabricated by attaching lithium sheets (Φ12mm) and LFP composite cathode (Φ12mm) to both sides of the CPEs. The assembled batteries were heated under 60°C for 12 hours to improve interfacial contact.

The charge/discharge tests of the cells were carried out using Land 2001A at 60°C. The electrochemical impedance spectroscopy (EIS), cyclic voltammetry (CV), linear sweep voltammetry (LSV), and potentiostatic polarization tests for lithium transference number measurement were conducted via a Biologic VSP electrochemical workstation.

For each CPE, the value of lithium transference number (t_{Li+}) can be calculated by Bruce-Vincent method given by the following formula.

$$t_{Li^+} = \frac{I_s(\Delta V - I_i R_i)}{I_i(\Delta V - I_s R_s)}$$

Where ΔV is a small DC bias applied to polarize the sample,

I_i is an initial value of current upon polarization with DC bias,

I_s is a current reached in the steady-state for sample polarized with DC bias,

R_i and R_s are resistances of solid electrolyte interface (SEI) before and after polarization, respectively.

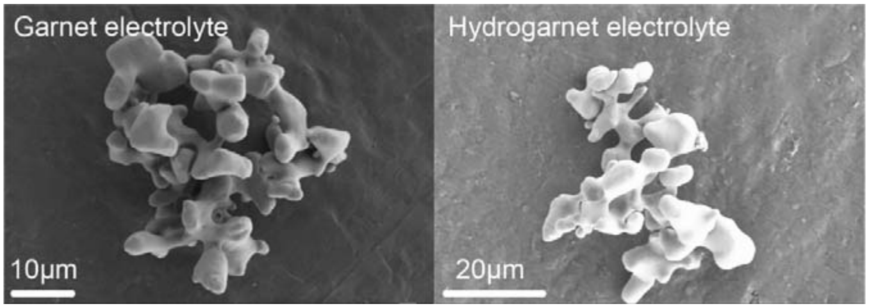

Figure S1: SEM images of both garnet and hydrogarnet electrolytes. After hydrothermal treatment under rationally selected conditions, no obvious alteration can be observed from SEM images.

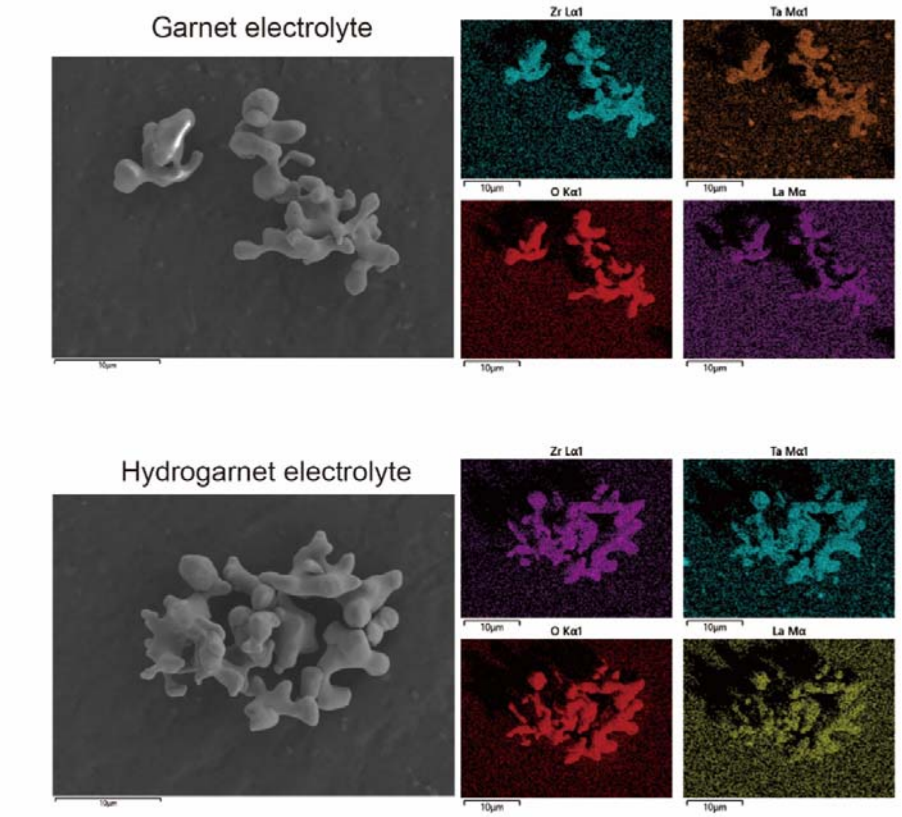

Figure S2: EDS-Mapping images of both garnet and hydrogarnet electrolyte particles. After hydrothermal treatment under rationally selected conditions, no element segregation can be observed from EDS results.

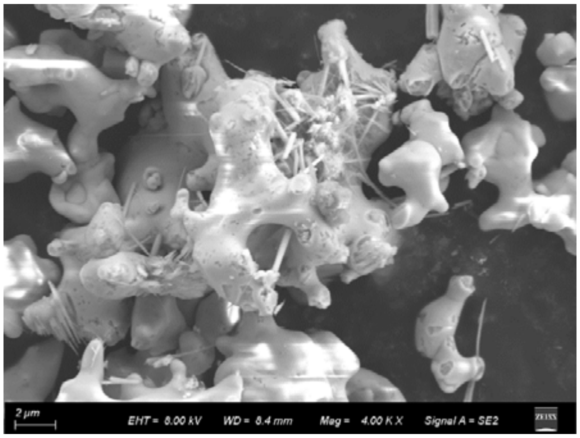

Figure S3: SEM image of the resultant powders after hydrothermal treating garnet electrolyte powder under a higher temperature (453 K). A great number of needle-like impurities can be observed from SEM images.

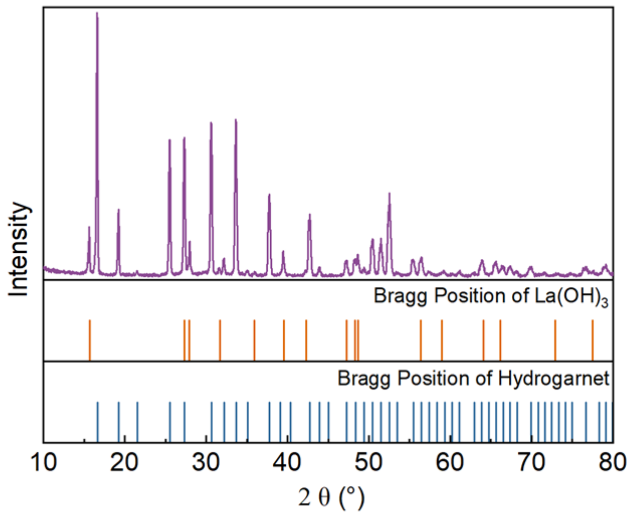

Figure S4: XRD pattern of the resultant powder illustrated in Figure S3, impurity peaks belonging to La $(OH)_3$ were observed in this XRD pattern, suggesting a La loss may happen under a higher temperature.

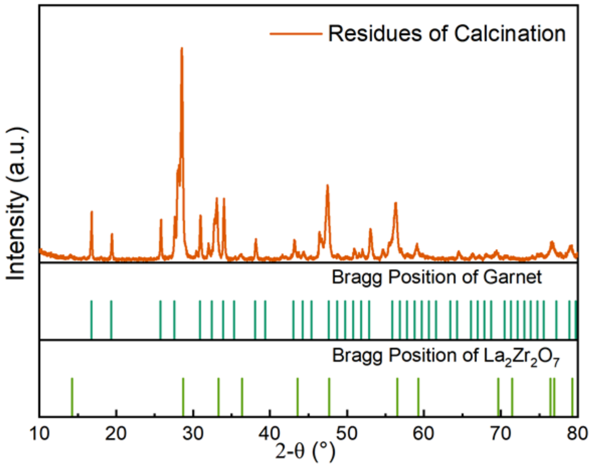

Figure S5: XRD pattern of the calcinated (960 °C for 3 h) hydrothermal treated garnet electrolyte (hydrogarnet) powder. Prominent peaks of $La_2Zr_2O_7$ are observed after calcination, suggesting the hydrothermally treated garnet powder will decompose to $La_2Zr_2O_7$ accompanied by proton loss in the form of H_2O.

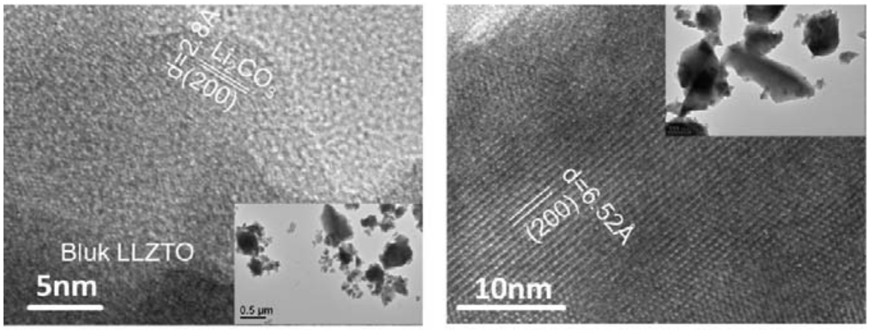

Figure S6: High-resolution TEM images of both garnet and hydrogarnet electrolyte powder, the measured d_{002} is 6.52 Å, in accordance with the lattice parameter (13.04 Å).

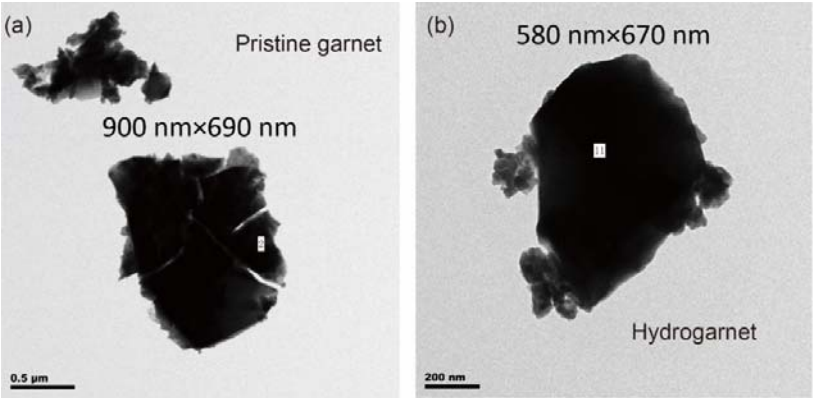

Figure S7: TEM images of the electrolyte particles selected for SEAD tests. Unsatisfying, in order to accumulate enough intensity of the characteristic spots of hydrogarnet, both garnet and hydrogarnet particles selected are sub-micron levers to accumulate sufficient intensity of the diffraction beam. As a consequence, double diffraction spots can be seen in both SEAD patterns obtained experimentally. Despite this, double diffraction spots of the $I a\bar{3}d$ structure will only have even miller indices, spots of (310) and (510) are also prohibited, ensuring the persuasiveness of SEAD results.

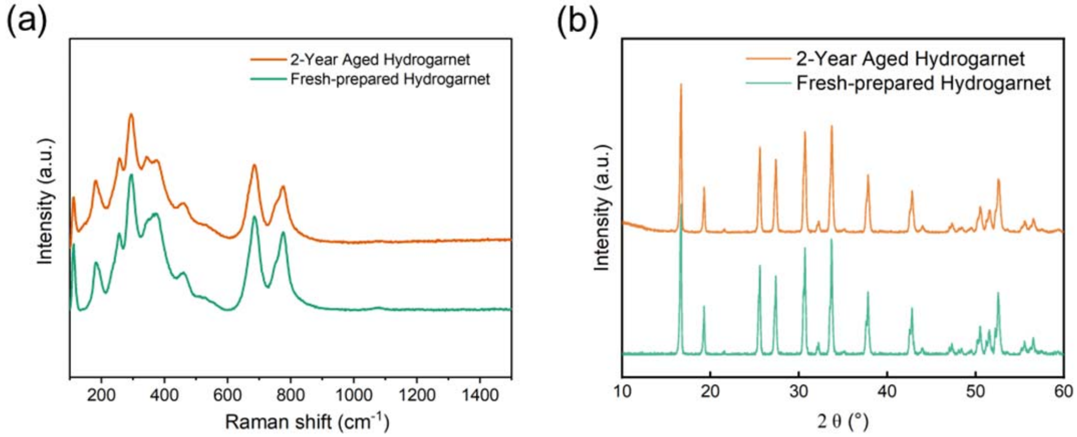

Figure S8: (a) Raman spectra and (b) XRD patterns of hydrogarnet before and after being aged in ambient air for 2 years. From both Raman spectra and XRD patterns, no obvious signal of Li_2CO_3 is observed in hydrogarnet powder even after a 2-year aging, in accordance with FT-IR results. Moreover, no obvious change happened to the hydrogarnet, indicating reliable phase stability of the hydrogarnet.

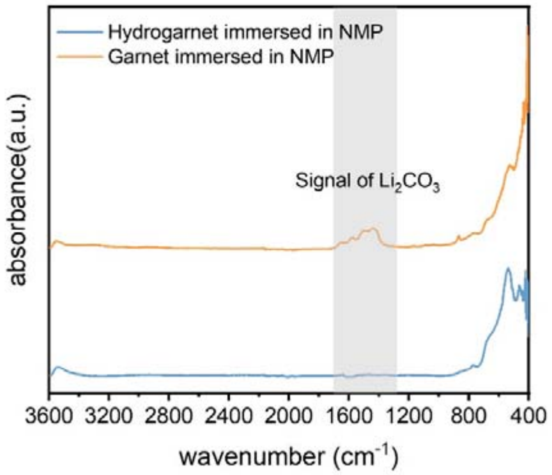

Figure S9: To confirm this prospect, both hydrogarnet and garnet powder were mixed with NMP solvent and heated for 6 hours under 80 °C. FT-IR spectra of the resultant powders were collected. The FT-IR spectra show that no obvious change of hydrogarnet is observed while prominent Li_2CO_3 is generated in garnet powder, suggesting an improved stability of hydrogarnet toward NMP solvent.

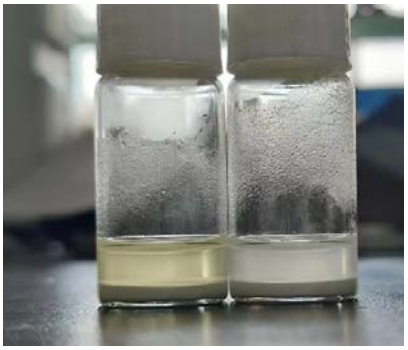

Figure S10: Optical photos of the NMP solvent after contact with both hydrogarnet (the right bottle) and garnet (the left bottle) powder. The color change suggests the harmful effect of garnet electrolyte on NMP solvent, while this kind of harmful effect is absent for hydrogarnet.

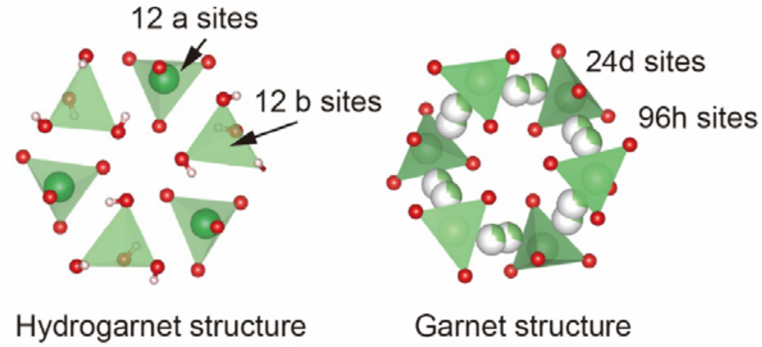

Figure S11: Detailed coordination environment (a six-member ring parallel to [111] axis) of lithium ions. In the initial garnet structure, both 24d sites and 96h sites are occupied by lithium ions. However, for the hydrogarnet structure, only lithium ions in 12a sites are retained.

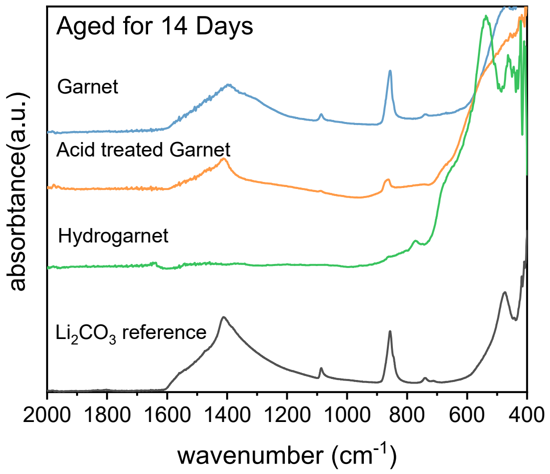

Figure S12: FT-IR spectra of garnet electrolyte, acid-treated garnet, and hydrogarnet electrolytes after 14 days' aging. From the FT-IR spectra, signal of Li_2CO_3, although weaker than garnet, can be seen in the aged acid-treated garnet electrolyte. When compared to hydrogarnet electrolyte (where signals of Li_2CO_3 are absent), the deficient stability of acid-treated garnet electrolyte is revealed.

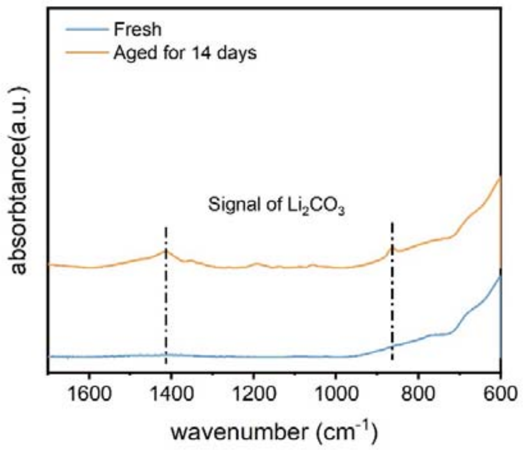

Figure S13: FT-IR spectrum of garnet electrolyte subjected to hydrothermal treatment under 100°C (Fresh) and the corresponding spectrum after 14 days' aging (Aged for 14 days). Signals of Li_2CO_3 can be observed here after aging and this instability may be due to the inadequate conversion from garnet to hydrogarnet.

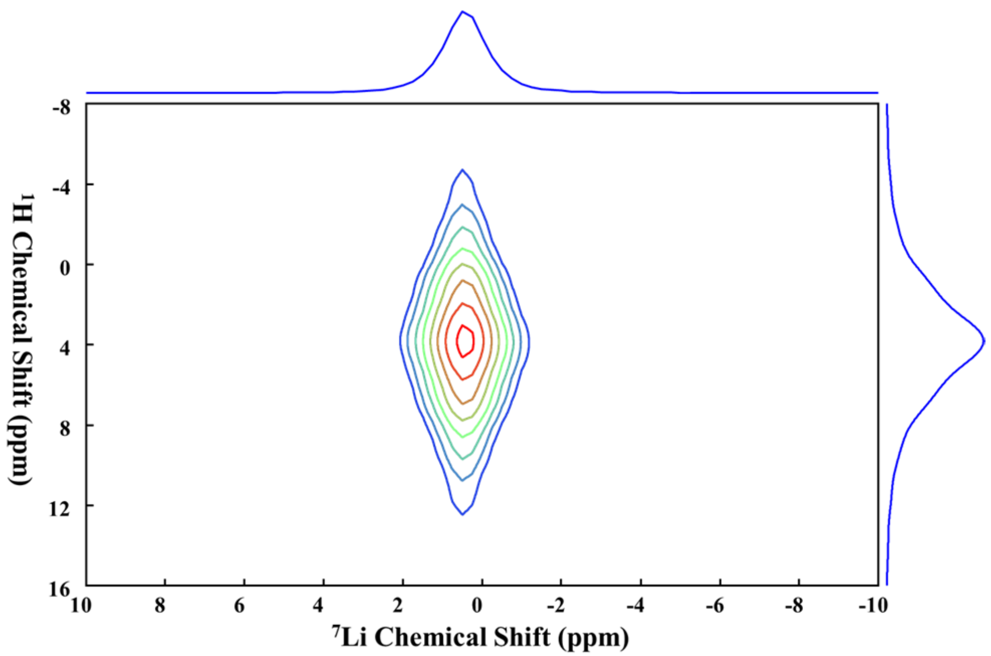

Figure S14. The ^{1}H-^{7}Li HETCOR MAS NMR result of hydrogarnet. There is only one single peak in both ^{1}H and ^{7}Li projection observed, implying that only one lithium site remained as well as one proton position generated in this hydrogarnet structure. The broad peak in ^{1}H projection centered at +4 ppm approximately represents the formation of hydroxyls according to some previous reports.[7] Moreover, in the ^{1}H-^{7}Li HETCOR MAS NMR result, the clear signal correlating the single ^{7}Li band with the ^{1}H band also supports the existence of lithium-hydroxyl interaction in the hydrogarnet structure.

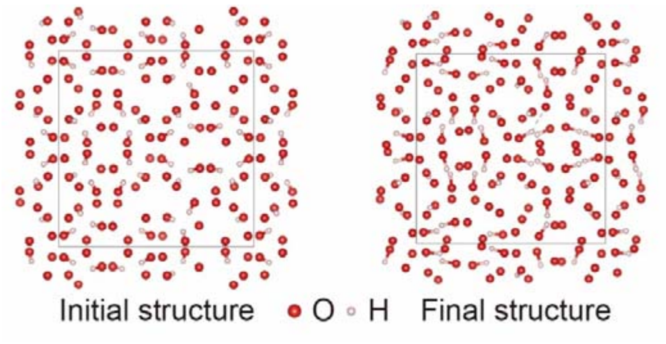

Figure S14: Schemes of proton positions inside hydrogarnet structure. To realize the distribution of proton and get the final crystalline structure, structural optimization was carried out using first-principal calculations (DFT). According to neutron powder diffraction (NPD) results obtained by Orera, Alodia et al.,[8] the initial positions of protons are set in 48g sites and anchored by oxygens. Results of the structural optimization also supported this structure except for longer O-H band distances.

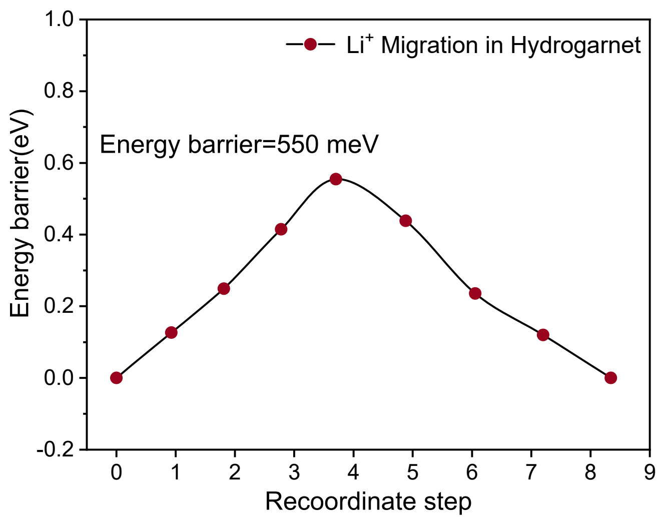

Figure S15: Lithium migration activation energy profile alone Y1-X2 route. The activation energy alone in this route passing through 4 protons is 550 meV.

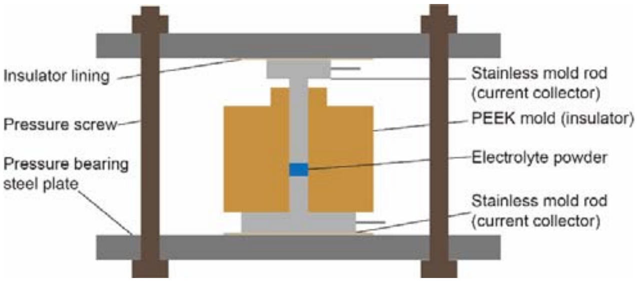

Figure S16: This scheme showed the cell configuration used for cold-pressed ionic conductivity measurement. After the cell was correctly assembled, the whole cell was placed under a manual pellet hydraulic press machine and a pressure of 6 tons was applied to this configuration (Φ12mm rods). Subsequently, we tightened the pressure screws, ensuring that the pressure was maintained even after the press machine was removed. Both PEEK mold and insulator linings were applied in this cell to isolate the anode and cathode. Then EIS tests were conducted on this cell under different temperatures.

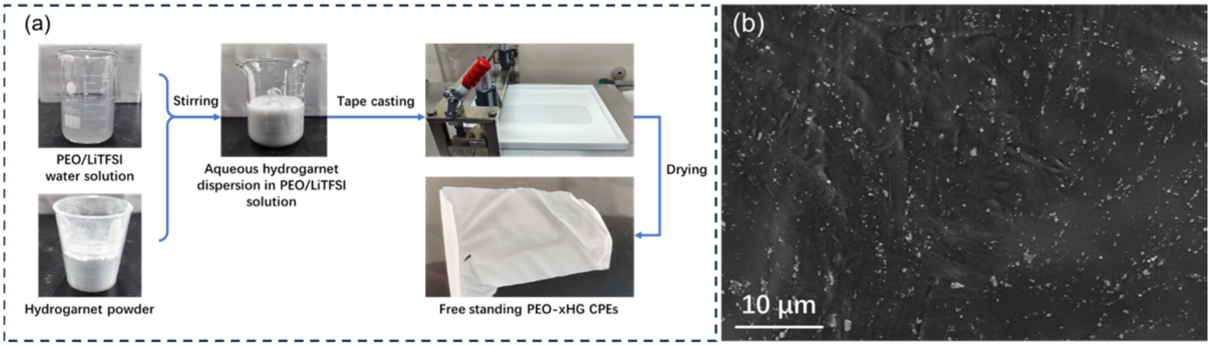

Figure S17: (a) A scheme demonstrating the process for preparing the PEO-xHG CPE based on the laboratory procedure. Consequently, free-standing PEO-xHG electrolyte membranes can be feasibly obtained; (b) SEM image of the PEO-20HG composite electrolyte, and the evenly distributed hydrogarnet particles in the PEO matrix can be observed.

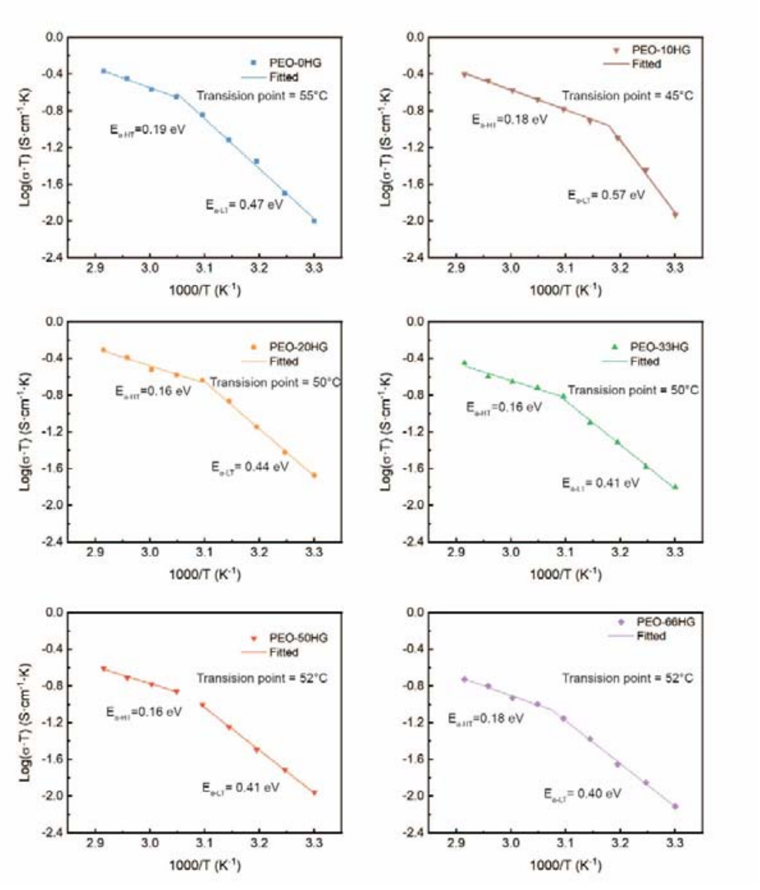

Figure S18: The Arrhenius plots of PEO-xHG (x=0, 10, 20, 33, 50, 66 wt.%) CPEs and the corresponding lithium-ion migration activation energies.

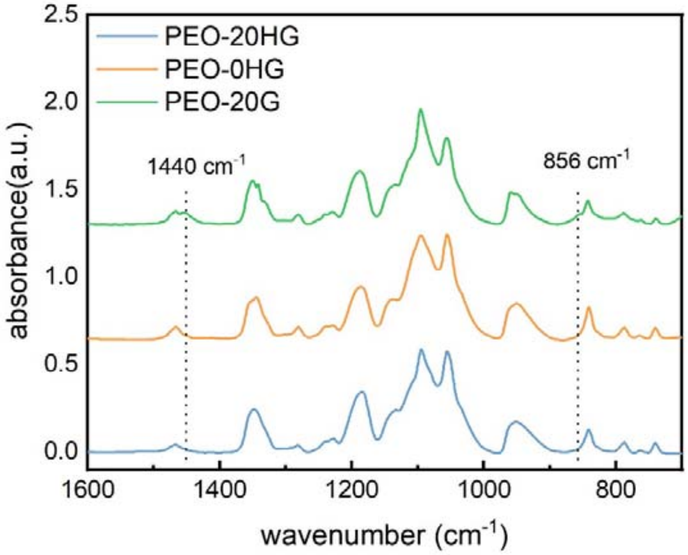

Figure S19: FT-IR spectra of PEO-20HG, PEO-0HG, and PEO-20G (containing 20 wt.% garnet electrolyte, fabricated using aqueous solution) CPEs. Two bands at 1440 cm^{-1} and 856 cm^{-1} belonging to Li_2CO_3 can be observed in PEO-G20 electrolytes while are absent in PEO-HG20 electrolytes.

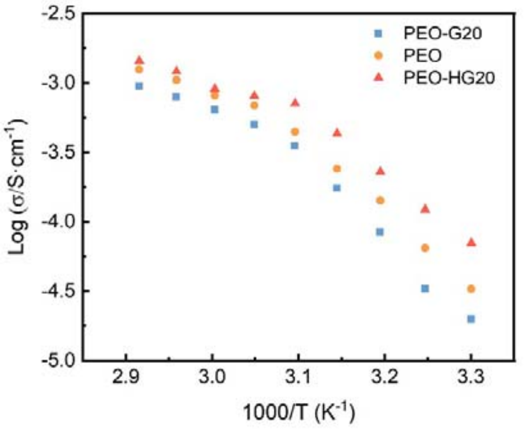

Figure S20: The ionic conductivity of PEO-20HG, PEO-0HG, and PEO-20G CPEs under different temperatures. It can be seen that PEO-G20 CPE possesses the lowest ionic conductivity in the whole temperature range and even lower than that of PEO-0HG electrolyte, revealing the adverse effects of impurities in PEO-G20 on ionic conductivity and the incompatibility of garnet electrolyte toward water-based fabrication.

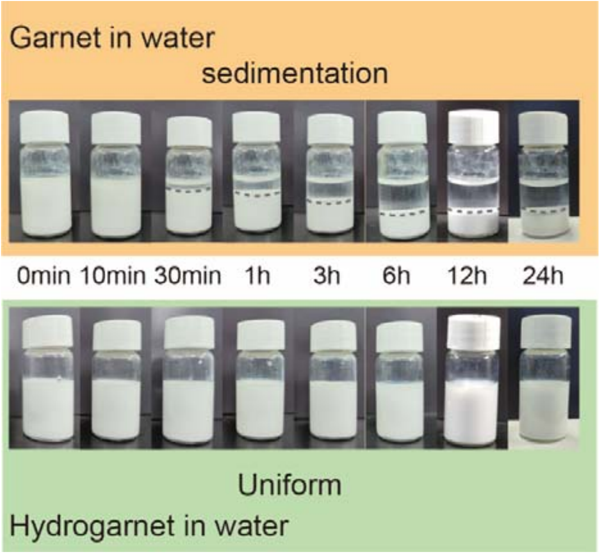

Figure S21: The stability of both garnet electrolyte powder and hydrogarnet electrolyte powder was tested by leaving the dispersion undisturbed for 24 hours and observing the sedimentation. As we can see from the photos, sedimentation was observed in the garnet dispersion after 30 minutes while hydrogarnet dispersion remained stable for nearly 24 hours.

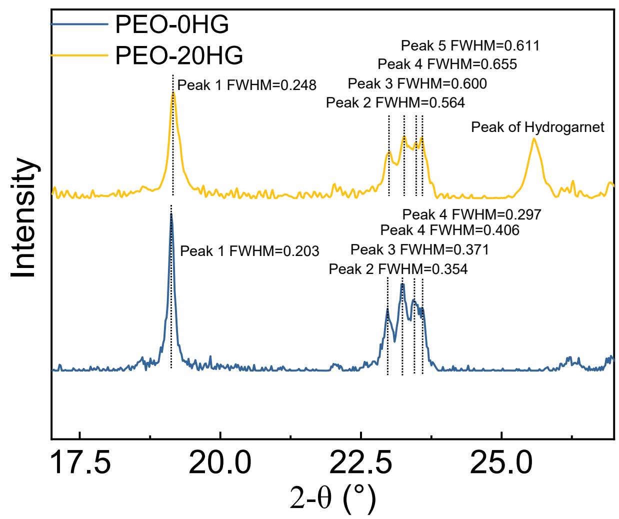

Figure S22: XRD patterns of PEO-0HG and PEO-20HG. FWHM values of the listed peaks of PEO-20HG electrolytes are larger than those of PEO-0HG electrolytes, revealing a lower crystallinity of PEO-20HG electrolytes.

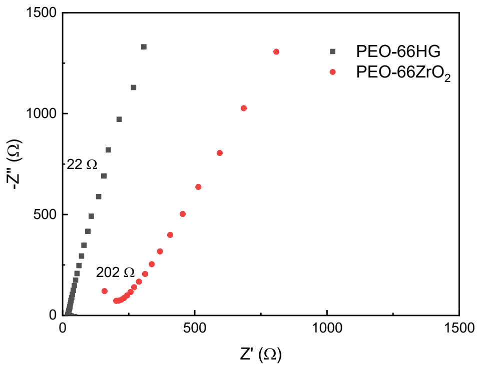

Figure S23: EIS plots of both PEO-66HG and PEO-66ZrO_2 CPEs. The impedance of PEO-66HG was 20 Ω (1.96 cm^2, 100 μm), corresponding to an ionic conductivity of 3.53 $\times 10^{-4}$ $S \cdot cm^{-1}$, while the impedance of PEO-66ZrO_2 was 202 Ω (1.96 cm^2, 100 μm), corresponding to a much lower ionic conductivity of 2.37 $\times 10^{-5}$ $S \cdot cm^{-1}$.

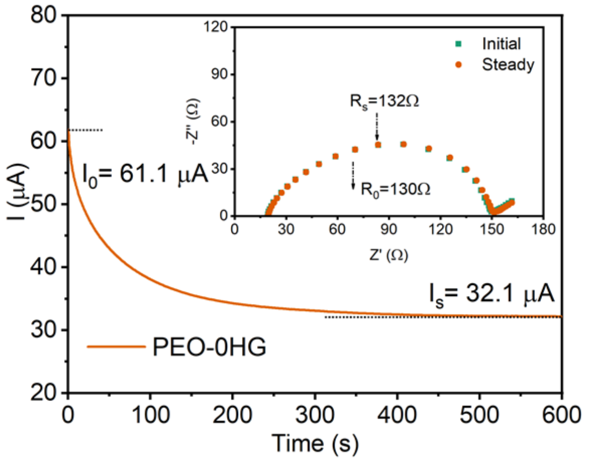

Figure S24: I-t curves of the Li-symmetry cell assembled PEO-20HG electrolyte before and after polarization (the insets showed the corresponding Nyquist plots).

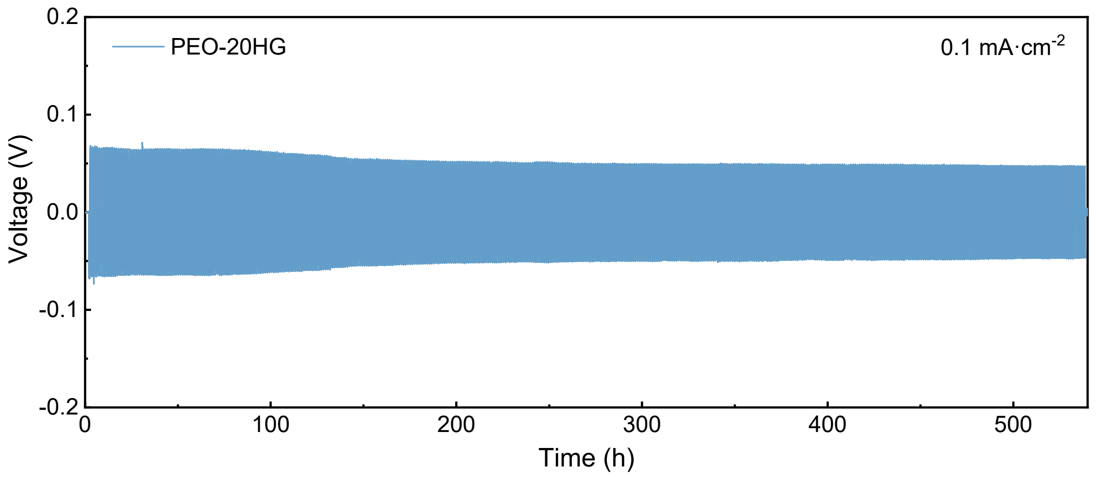

Figure S25: Performance of lithium symmetry cells assembled with PEO-20HG CPE at a current density of 0.1 mA·cm^{-2} under 50 °C. Cell with PEO-20HG CPE can operate stable for more than 525 hours with an overpotential of 45 mV.

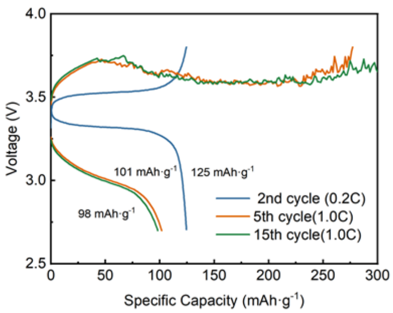

Figure S26: The charge and discharge curves of Li-LFP batteries assembled with PEO-20G CPE at different cycles. At the first 3 cycles at 0.2 C, the Li-LFP battery with PEO-0HG CPE demonstrated a relatively stable operation with a low specific capacity of 125 mAh·g^{-1}. However, when the charge rate increased to 1.0C, PEO-20G started to decompose severely.

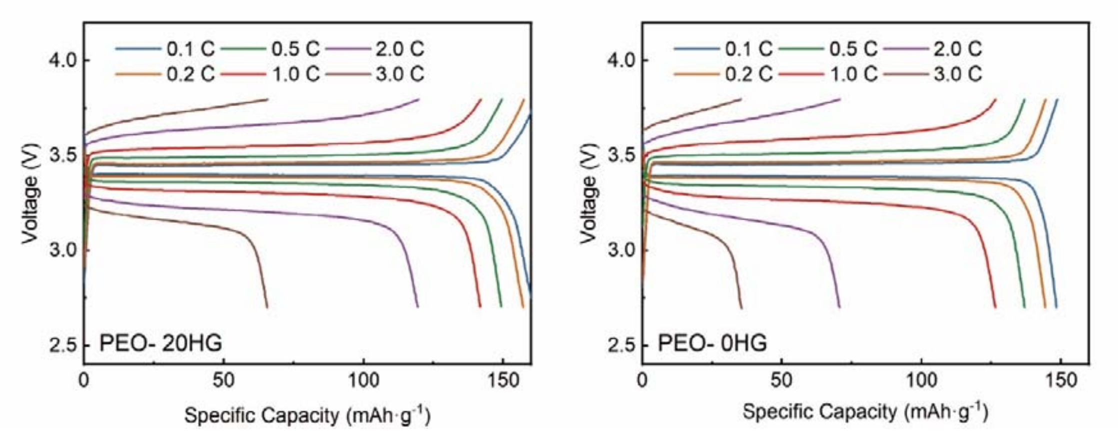

Figure S27: The charge and discharge curves of Li|LFP batteries assembled with both PEO-0HG CPE and PEO-20HG CPE under different rates at 50 °C. Batteries with PEO-20HG demonstrated lower overpotentials and higher reversible capacities under different rates.

Table S1：ICP-OES test results of both garnet and hydrogarnet electrolyte.

Sample	La content (wt.%)	Li content (wt.%)	Zr content (wt.%)	Ta content (wt.%)
**Garnet (ideal)**	47.3	5.12	15.5	10.26
**Garnet**	39.9	4.75	11.0	7.4
**Hydrogarnet**	38.0	1.08	11.9	7.9

The first line showed the content of metal elements calculated from the chemical formula of the garnet electrolyte. Although some content deviation was observed, the Li/La, Li/Zr, and Zr/Ta ratios remained the same between the calculated results and the experimental results. Observed in the ICP results, Li content in hydrogarnet dropped to 22.7% of the pristine garnet, corresponding to the change from 6.5 lithium per garnet formula to 1.5 lithium per hydrogarnet formula.

It should be noted that, based on the TG results, a similar Li/H ratio can also be obtained by the following equation.

$$\mathrm{Li_{6.5-\mathit{x}}La_3Zr_{1.5}Ta_{0.5}O_{12-\mathit{x}}OH_{\mathit{x}}} \triangleq \mathrm{Li_{6.5}La_3Zr_{1.5}Ta_{0.5}O_{12}} + \mathrm{La_2Zr_2O_7} + \frac{x}{2}\,\mathrm{H_2O}$$

$Ratio_{Mass\ loss} = \frac{xM_{H_2O}}{2M_{hydrogarnet}} = \frac{18x}{881-5.93x} = 5.1\%$, x=4.9.

Table S2: The intensity of X-ray scattering and electron beam scattering for the hydrogarnet electrolyte of different lattice planes at the d range of 5.31 Å to 1.84 Å.

**Lattice plane (hkl)**	**d (Å)**	**2θ (°)**	**Intensity of X-ray scattering factor(%)**	**Intensity of electron beam scattering factor(%)**
(211)	5.31	16.70	98.96127	22.88668
(220)	4.60	19.31	27.75361	9.507223
**(310)**	**4.11**	**21.62**	**0.002232**	**0.021016**
(321)	3.47	25.64	64.78953	12.02259
(400)	3.25	27.44	61.20819	100
(420)	2.91	30.76	90.96847	49.79569
(332)	2.77	32.30	5.269616	0.50487
(422)	2.65	33.78	100	42.04526
(510)	2.55	35.20	0.001277	0.000566
(431)	2.55	35.20	3.210822	2.499089
(521)	2.37	37.91	44.081	19.04195
(440)	2.30	39.20	0.958321	0.0172
(530)	2.23	40.46	0.004057	0.016925
(532)	2.11	42.88	26.15837	12.21598
(611)	2.11	42.88	18.90804	20.57743
(620)	2.06	44.06	8.052442	7.006056
(541)	2.01	45.20	0.088531	0.130257
(631)	1.92	47.43	8.476667	1.858532
(444)	1.88	48.52	4.639298	18.52591
(543)	1.84	49.59	0.143715	0.261205
(710)	1.84	49.59	0.013017	0.117631

It can be seen that for the lattice plane (310), the scattering factor of electron beams is an order larger than that of X-rays.

Reference

[1] Y. Li, Y. Cao, X. Guo, *Solid State Ion.* **2013**, 253, 76.

[2] J. P. Perdew, K. Burke, M. Ernzerhof, *Physical Review Letters* **1996**, 77, 3865.

[3] G. Kresse, D. Joubert, *Phys. Rev. B* **1999**, 59, 1758.

[4] G. Henkelman, H. Jónsson, *J. Chem. Phys.* **2000**, 113, 9978.

[5] M. Capdevila-Cortada, Z. Łodziana, N. López, Vol. 6, ACS Publications, 2016, 8370.

[6] a) I. Brown, D. Altermatt, *Acta. Crystallogr. B. Struct. Sci. Cryst. Eng. Mater.* **1985**, 41, 244; b) I. D. Brown, *The chemical bond in inorganic chemistry: the bond valence model*, Oxford university press, **2016**.

[7] a) G. Larraz, A. Orera, J. Sanz, I. Sobrados, V. Diez-Gómez, M. L. Sanjuán, *J. Mater. Chem. A* **2015**, 3, 5683; b) H. Munakata, T.-r. Koyama, T. Yashima, N. Asakawa, T. O-Nuki, K. Motokura, A. Miyaji, T. Baba, *J. Phys. Chem. C* **2012**, 116, 14551; c) G. Mogilevsky, C. J. Karwacki, G. W. Peterson, G. W. Wagner, *Chemical Physics Letters* **2011**, 511, 384; d) T. Isobe, T. Watanabe, J. B. d'Espinose de la Caillerie, A. P. Legrand, D. Massiot, *Journal of Colloid and Interface Science* **2003**, 261, 320.

[8] A. Orera, G. Larraz, J. A. Rodríguez-Velamazán, J. Campo, M. L. Sanjuán, *Inorg. Chem.* **2016**, 55, 1324.
